# Readiness of Primary Hospitals in Providing Neonatal Intensive Care Services in Ethiopia

**DOI:** 10.4314/ejhs.v31i2.15

**Published:** 2021-03

**Authors:** Hailemariam Segni Abawollo, Zergu Tafesse Tsegaye, Binyam Fekadu Desta, Ismael Ali Beshir

**Affiliations:** 1 JSI/USAID Transform: Primary Health Care Activity, Addis Ababa, Ethiopia

**Keywords:** Neonatal intensive care, NICU, Newborn care, Neonatal care, Primary hospital

## Abstract

**Background:**

The Ethiopian neonatal mortality has not shown much progress over the years. In light of this, the country has introduced interventions such as the utilization of newborn corners and neonatal intensive care units to avert preventable neonatal deaths. This study was conducted to assess readiness of primary hospitals in providing neonatal intensive care services.

**Methods:**

A health facility based cross-sectional study design was employed where data were collected using both prospective and retrospective techniques using a format adapted from national documents. SPSS version 25 was used for data entry and analysis using descriptive statistics.

**Results:**

Data were collected from 107 of 113 (94.7%) primary hospitals due to inaccessibility of some primary hospitals. The minimum national standard requirement of a level one neonatal intensive care unit for infrastructure was met by 63% (68/107) and 44% (47/107) had fulfilled the requirements for kangaroo mother care units. The average number of neonatal intensive care unit trained nurses per primary hospital was 2.6, 0.8 for general practitioners and 2.9 support staff; all of which is less than the minimum recommended national standard. The minimum national requirement for medical equipment and renewables for primary hospital level was fulfilled by 24% (26/107) of the hospitals, 65% (70/107) for essential laboratory tests, and 87% (93/107) for clinical services and procedures. The average number of admissions during the six months prior to the data collection was 87.2 sick newborns per facility with a ‘discharged improved’ rate of 71.5%, referral out rate of 18.4% and level one neonatal intensive care unit death rate of 6.6%. The remaining newborns had either left against medical advice or were still undergoing treatment during data collection.

**Conclusions:**

The overall readiness of primary hospitals to deliver neonatal intensive care services in terms of infrastructure, human resource, medical equipment, and laboratory tests was found to be low. There is a need to fill gaps in infrastructure, medical equipment, renewables, human resource, laboratory reagents, drugs and other supplies of neonatal intensive care units of primary hospitals to garner better quality of service delivery.

## Introduction

Globally, 2.7 million newborns die during the first month of life as a result of birth asphyxia, complications of preterm birth and infections. An additional 2.6 million babies are stillborn. The majority of the neonatal deaths and stillbirths (99%) occur in low and lower-middle-income countries with half of the deaths happening at home. Good-quality antenatal care (ANC), skilled care at delivery, and postnatal care (PNC) are crucial for the prevention of stillbirths and newborn deaths. About 75% of neonatal deaths could be avoided with simple, low cost tools that already exist within systems such as antibiotics for pneumonia and sepsis, sterile blades to cut umbilical cords and using knit caps and kangaroo care to keep babies warm (1).

In Africa, approximately 1 million babies are stillborn each year, of whom at least 300,000 die during labor. A further 1.16 million babies die in their first month of life and half of them, on the first day of their life. Another 3.3 million children will die before they reach their fifth birthday. Four million low birth weight babies and babies with neonatal complications will live but may not reach their full potential (2).

The national neonatal mortality has not shown progress as indicated by the 2019 mini EDHS, even a slight increment was observed from the 2016 rate of 29/1,000 live births (LBs) to 30/1,000 LBs in 2019. This may indicate that either the proposed strategies and interventions were not well implemented, or the strategies are not helping to meet the intended objectives and targets (3).

The major contributors to neonatal mortality are early neonatal deaths, i.e. within the first 7 days of life, which can be prevented through the utilization of good quality essential newborn care (ENC) services immediately after delivery on top of quality obstetric care. The proportion of health facilities in the country delivering hygienic cord care was 52%, immediate and exclusive breast feeding 53% and thermal care 52% with mean availability of newborn signal functions at 38% (4). The neonatal mortality rate of the country is disproportionally high accounting to 44% of under-five deaths (5).

To reduce the stagnating neonatal mortality, the country has put in place various interventions which include but are not limited to: establishment of newborn corners to provide ENC services in health facilities which are mandated to conduct skilled delivery and establishment of different levels of neonatal intensive care units (NICU) with kangaroo mother care (KMC) centers in hospitals. NICUs are setup to provide advanced care for sick newborns that require more specialized care and attention (6).

An NICU or Intensive Care Nursery (ICN) is an intensive care unit (ICU) specializing in the care of ill/sick or premature newborn infants which are likely to die as a result of simple conditions that can be easily prevented (7).

In Ethiopia, the levels of NICU care, expected to be provided by different types of hospitals vary. Primary hospitals (PHL) are expected to have a minimum of level one NICUs, regional referral hospitals should at least have level two NICUs, and specialized teaching hospitals must have level three NICUs (6).

A cross-sectional study conducted in Debrebirhan Hospital indicated that the availability of all the required infrastructure, equipment, trained manpower and supplies is key to providing quality neonatal health services in hospitals. Additionally, the proper utilization of available resources was also found to be essential (8).

USAID Transform: Primary Health Care project which began in January 2017 supports primary hospitals of four regions (Amhara, Oromia, South Nations Nationalities and Peoples/SNNP, and Tigray) where level one NICUs are found. The project has been supporting NICUs of 113 PHLs through capacity enhancement of NICU nurses (one-month long training at tertiary hospitals), one-week orientation for general practitioner (GP) physicians, NICU medical equipment maintenance, and technical support to NICUs during follow-up and supportive supervision visits by project staffs.

The aim of this study was to assess primary hospitals' current status of readiness to provide neonatal intensive care services by assessing the status of infrastructure, human resource, medical equipment, laboratory tests and clinical services in hospitals.

## Materials and Methods

**Study setting and period:** The project works in four regions of the country (Tigray, Amhara, Oromia, and South Nations Nationalities and peoples/SNNP). These regions are divided into clusters of which four are in Tigray, nine in Amhara, ten in Oromia, and six in SNNP. The project is run by country office staffs in Addis Ababa, regional office staffs in capital cities of the mentioned four regions, and cluster office staffs working in zonal health departments of the zones in those four regions.

The project's interventions focus on primary level of healthcare which includes PHLs and their catchment health centers (HC). Within the project's intervention areas, there are 113 PHLs which are expected to have level one NICUs. The assessment was conducted from January 1 to March 31, 2020.

**Study design**: A health facility based cross-sectional study design with both prospective and retrospective data collection technique was used to conduct this assessment.

**Study population**: This assessment focused on checking the current status of NICUs in the 113 intervention PHLs in terms of status of infrastructure, human resource, availability of essential medical equipment and renewables, availability of laboratory tests, and status of clinical services rendered including procedures performed.

**Sample size and sampling**: The assessment was conducted at all the intervention PHLs within the project catchment areas.

**Materials**: A national NICU status assessment tool was adapted and used by incorporating additional minimum requirements for level one NICU from respective national documents (6,13). The tool was organized in a simplified excel sheet for data collection purpose.

**Data collection**: The project's cluster staffs were data collectors at their respective catchment PHLs. The data collection was conducted through integration with routine follow-up visits as the cluster staffs are expected to conduct one round of follow-up visit to each of their respective PHLs every three months.

The number of project staffs involved in data collection per cluster varies from three to five, based on the size of the catchment area of a specific cluster. The data collectors were given orientation on the assessment protocols including the tools by the investigators. Data quality was checked by regional maternal and newborn health (MNH) officers, who possess a master's degree in public health with backgrounds in midwifery, on daily bases that would identify gaps, if any, and address them immediately at the field level. The data were collected electronically and each of the clusters sent the collected data to regional MNH officers. The regional MNH officers then sent the data to the country office after finalizing the data collection.

**Data analysis**: Data were cleaned by investigators and data entry was carried out by a data entry clerk. Data analysis was conducted at country office level by the investigators using statistical software SPSS version 25.0. Descriptive statistics was used to analyze and report findings.

**Ethics**: Ethical clearance was obtained from John Snow Incorporated (JSI) institutional review board (IRB), reference number IRB #20-17E. Each of the PHL leaders and responsible heads of the NICUs were handed an information sheet and copy of the ethical clearance letter from JSI IRB and were asked for verbal consent to go ahead with the assessment of their respective PHL NICUs. The NICU professionals who gave information/data during the data collection were also asked for verbal consent.

## Results

Data from 107 of the 113 PHLs (94.7%) were collected and the findings of the assessment are categorized into five major pillars for NICU service delivery. Six of the primary hospitals were not accessible during data collection time.

**Infrastructure**: Based on the national minimum infrastructure standard expected at level one NICU, (6), 63% (68/107) of the NICUs fulfilled the minimum requirements ranging from 38% (41/107) for separate ‘Boiling and autoclaving service area’ to 88% (94/107) for ‘NICU's location is adjacent to the delivery room’ (Table 1).

**Table 1:** Status of infrastructure at NICU of PHLs, USAID Transform: Primary Health Care, January- March 2020 (N=107)

Variable	Number	Percent
Infrastructure	68	63%
Location adjacent to delivery room	94	88%
The NICU has direct access to the hospital's transport receiving area	80	75%
Service units are connected to each other allowing transport of newborns	83	78%
without being exposed to outside cold weather		
Room size: 8–12 square meters	89	83%
Gowning area at the entrance	72	67%
Hand washing stations	62	58%
Examination area	66	62%
Clean area for mixing IV fluids and medications	80	75%
Mothers' area for expression of breast milk, BF and learning mother crafts	43	40%
Boiling and autoclaving	41	38%
General support area	56	52%
Procedure room	49	46%

Kangaroo mother care (KMC) service is available in 85% (91/107) of the NICUs. Forty-four percent (47/107) of them had KMC service delivery units fulfilling the minimum national standard for KMC of level one NICUs. The availability of the national minimum standard materials in the KMC units ranged from 29% (31/107) for ‘At least four KMC beds’ to 75% (67/107) for ‘Availability of chairs’ within the KMC units (Table 2).

**Table 2:** Status of KMC infrastructure at NICU of PHLs, USAID Transform: Primary Health Care, January-March 2020 **(N=107 for first two variables, N=89 for the rest three variables)**

Variable	Number	Percent
KMC	47	44%
4 KMC beds	31	29%
Availability of toilet and shower	36	40%
Availability of table	55	62%
Availability of chairs	67	75%

**Human resource**: The national recommended number of NICU trained nurses per a 12-bedded level one NICU is at least four, of GPs is one to two, support staffs is four (6). The average number of NICU trained nurses per NICU at the project site was 2.6, 0.8 GPs, and 2.9 support staffs - all of which are less than the minimum national standard recommendations (Table 3).

**Table 3:** Status of human resource at NICU of PHLs, USAID Transform: Primary Health Care, January-March 2020 (N=107)

Variable	
	
Human resource	Average number
Average # of staff Nurses (Working in NICU)	3.2
Average # of NICU trained nurses	2.6
Average # of GP with 4–5 days training/orientation	0.8
Average # of health officers with NICU training	0.3
Average # of support staff: Porters	0.8
Average # of support staff: Cleaners	2.1

**Essential medical equipment and renewables**: The national minimum standards for essential medical equipment and renewables in level one NICUs are available in 24% (26/107) of the NICUs. The availability of these medical equipment and renewables ranged from 0% for ‘Hand operated 250 ml neonatal resuscitator’ (at least 10 functional), ‘Neonatal laryngoscope set’ (at least 2 functional) and ‘Bubble CPAP which has a compressor’ (at least 4 functional) to 85% (91/107) for ‘Biannual neonatal stethoscope’ (at least one functional) (Table 4).

**Table 4:** Availability of essential medical equipment and renewables at NICU of PHLs, USAID Transform: Primary Health Care, January-March 2020 (N=107)

Variable	Number	Percent
Essential medical equipment and renewable	26	24%
Warmer system, new-born, radiant W/access-S0002065: at least 2 functional	8	7%
NICU infection prevention consumables: at least 1 functional	90	84%
Radiant warmer to warm the room: at least 2 functional	23	21%
Oxygen Cylinder 20 liter: at least 1 functional	81	76%
Photo therapy unit, w/access S0002032 and S0002018 irradiance meter: at least 2	8	7%
functional		
Neonatal resuscitator, hand operated, 250 ml: at least 10 functional	0	0%
Laryngoscope set, neonate: at least 2 functional	0	0%
Pump, suction, portable-s0002028: at least 2 functional	9	8%
Suture set: at least 2 functional	15	14%
IV infusion pump (Device used to deliver fluids into a patient's body in a	30	28%
controlled manner): at least 1 functional		
Oxygen hood, S and M, set of 1 each, including connecting tubes: at least 2	6	6%
functional		
NICU Bed: at least 12 functional	2	2%
Standard adult hospital beds with mattress: at least 10 functional	23	21%
Oxygen concentrator and flow splitter: at least 2 functional	25	23%
Incubator: at least 2 functional	29	27%
Glucometer: at least 1 functional	61	57%
Thermometer, clinical, digital, 32–42 °C: at least 1 functional	79	74%
Weighing Scale neonate (Digital): at least 2 functional	20	19%
Pulse Oximeter, bedside, neonatal: at least 2 functional	10	9%
Stethoscope, biannual, neonatal: at least 1 functional	91	85%
Examination Lamp/Light, mobile: at least 2 functional	11	10%
Tape measure, vinyl-coated, 1.5m: at least 1 functional	67	63%
Basin, kidney, stainless steel, 825 ml: at least 2 functional	24	22%
Tray (dressing set): at least 2 functional	16	15%
Infusion stand: at least 12 functional	1	1%
Bubble CPAP which has a compressor: at least 4 functional	0	0%
LP set: at least 2 functional	1	1%
Infant meter, plexi, 105 cm: at least 1 functional	38	35%
Embrace for KMC/KFC: at least 2 functional	8	7%
Computer: at least 1 functional	16	15%
LED TV for health education: at least 1 functional	4	4%

**Laboratory tests**: The national minimum standard essential laboratory tests for level one NICUs were available in 65% (70/107) of the NICUs. The availability of these tests ranged from 12% (13/107) for ‘Culture and sensitivity of any body fluid’ to 99% (106/107) for ‘Blood group and Rh’ status determination (Table 5).

**Table 5:** Availability of essential laboratory tests at NICU of PHLs, USAID Transform: Primary Health Care, January-March 2020 (N=107 for the first two variables, N=106 for the rest of the variables)

Variable	Number	Percent
Laboratory tests	70	65%
CBC (any of WBC & Diff, RBC, Hgb, HCT, Platelet count)	85	79%
Blood Morphology	40	37%
Blood Film	101	94%
Bleeding time & Coagulating time	34	32%
ESR	84	78%
Reticulocyte count	48	45%
Blood group & Rh	106	99%
VDRL	103	96%
Blood Chemistry (any of SGOT, SGPT, Bilirubin direct & total, BUN)	45	42%
Serum electrolytes (any of Sodium, Potassium, Chloride, Phosphorous, Calcium)	27	25%
HBsAg	94	88%
Gram stain	60	56%
Urine analysis	104	97%
Stool exam	104	97%
Culture and sensitivity of any fluid	13	12%

**Clinical services and procedures**: The national minimum standard clinical services and procedures expected to be available at level one NICUs were present in 87% (93/107) of the NICUs. The availability of these clinical services and procedures ranged from 18% (19/107) for ‘Lumbar puncture (LP)’ to 99% (106/107) for ‘Insertion of nasogastric tube’ (Table 5).

The average number of admissions to the 107 level one NICUs during the six months prior to data collection was 87.2 sick newborns per NICU. The average ‘improvement and discharge’ rate was 71.5%, ‘referral out’ rate was 18.4%, and the rate for ‘deaths in NICU’ was 6.6%. The remaining had either left against medical advice or were still undergoing treatment during data collection. The average number of days of service interruption during the same period was 0.2 days per NICU.

## Discussion

In Ethiopian, basic newborn care unit (level one NICU) is a neonatal unit of a PHL that provides care to all sick newborns except those requiring assisted ventilation or major surgery (6).

NICUs should be located adjacent to delivery rooms and should have direct access to the hospital's transport receiving area, allowing transport of newborns within the hospital without using public corridors. Each newborn room is expected to have a minimum 8–12 m^2^ of clear floor space excluding hand washing stations and columns with adequate space for ancillary (supplementary) services (6).

Around two-thirds (63%) of the NICUs in this assessment fulfilled the minimum national standards in infrastructure. This low percentage contributes to the delivery of low-quality neonatal intensive care services. Similar findings were reported in other local studies and in studies conducted in other countries within Africa (9,10).

Level one NICUs need to have a KMC unit, with the minimum size that can accommodate four adult beds with a toilet and a shower, a table, and chairs (6). The proportion of NICUs delivering KMC services in this assessment was 85%, but KMCs with the minimum required space and materials was low (44%) which may be due to the low proportion of KMCs with the minimum room size and availability of a toilet and a shower.

Successful provision of quality services by a NICU depends not only on the equipped unit but also on the availability of round-the-clock clinical expertise, backed up by monitoring devices and equipment. Thus, the unit should have the required number of appropriately trained and qualified nurses, physicians and support staffs (6). The average number of NICU trained nurses, GPs and other support staffs in this assessment was found to be lower than the required numbers to run a level one NICU and is a critical obstacle to delivering quality care to newborns in the NICUs. Other studies also have shown nationwide shortages of adequate and well-trained health professionals contributing to low quality services (8,9,10,11).

In this assessment, 24% of the NICUs had the minimum required medical equipment and renewables for level one as per the national standard (6). Three of the items ['Hand operated 250 ml neonatal resuscitator’ (at least 10 functional), ‘Neonatal laryngoscope set’ (at least 2 functional) and ‘Bubble CPAP which has a compressor’ (at least 4 functional)] were not available in all the 107 NICUs and most of the items on the list were available in less than half of the NICUs. Other studies reported shortages of medical supplies, equipment, and essential medications as widespread problems in health facilities of the country, stating as often unavailable, broken, or inappropriate for use (8, 9). Another study finding reported that less than half of facilities had most of the supplies and equipment needed for newborns (12).

PHLs with level one NICUs should have laboratory test services listed in table 5 as it directly affects the quality of services provided at the level (6). Two-thirds (65%) of the NICUs in this assessment deliver those laboratory test services with six out of the 15 tests (40%) being delivered in less than half of the NICUs. Other studies also have reported similar problems with availability of necessary laboratory services to render quality NICU services (8).

Clinical services provided at level one NICUs are care at birth including resuscitation of asphyxiated newborns, managing sick newborns, postnatal care, follow up of high risk newborns, KMC services, referral services and linkage to immunization services, and procedures like LP, insertion of nasogastric tube (NGT), gastric lavage, dressing, wound care and stitching (6). This assessment showed that the majority (87%) of the project intervention NICUs are delivering the minimum expected clinical services and procedures except LP (18%) service which may be due to lack of the necessary equipment and renewables to deliver the service.

In conclusion, majority (87%) of the NICUs are delivering the minimum services expected to be delivered at level one NICUs, less than two-thirds (63%) of the project intervention NICUs have the minimum recommended infrastructure for level one NICUs, less than half (44%) of KMCs of the project intervention NICUs have the minimum recommended KMC infrastructure, the available necessary human resource per NICU at the project intervention facilities is far less than the minimum recommended for level one NICUs, less than a quarter (24%) of the NICUs have the minimum recommended medical equipment and renewables available for service delivery, and two-thirds (65%) of the project intervention NICUs are delivering the minimum standard laboratory tests required at level one NICUs.

Given the gaps this assessment has shown, there is a need for investment to improve the infrastructure, human resource, medical equipment, renewables, drugs, laboratory reagents and other supplies for a better quality NICU service delivery at the PHL level.

## Figures and Tables

**Table 6:** Availability of minimum clinical services and procedures at NICU of PHLs, USAID Transform: Primary Health Care, January-March 2020 (N=107)

Variable	Number	Percent
Clinical services and Procedures	93	87%
Care at birth including resuscitation of new-born	105	98%
Managing sick new-born	105	98%
Postnatal care	105	98%
Follow up of high-risk new-born	102	95%
Diagnose and refer surgical conditions	103	96%
Linked to immunization services	100	93%
Lumbar puncture	19	18%
Insertion of Nasogastric Tube	106	99%
Gastric lavage	97	91%
Dressing	96	90%
Stitch	89	83%
